# Supplementary figures and images for: Echocardiographic left ventricular stroke work index: An integrated noninvasive measure of shock severity
Source: PLoS One. 2022 Mar 9;17(3):e0262053. doi: 10.1371/journal.pone.0262053 (PMC8906587; doi:10.1371/journal.pone.0262053)

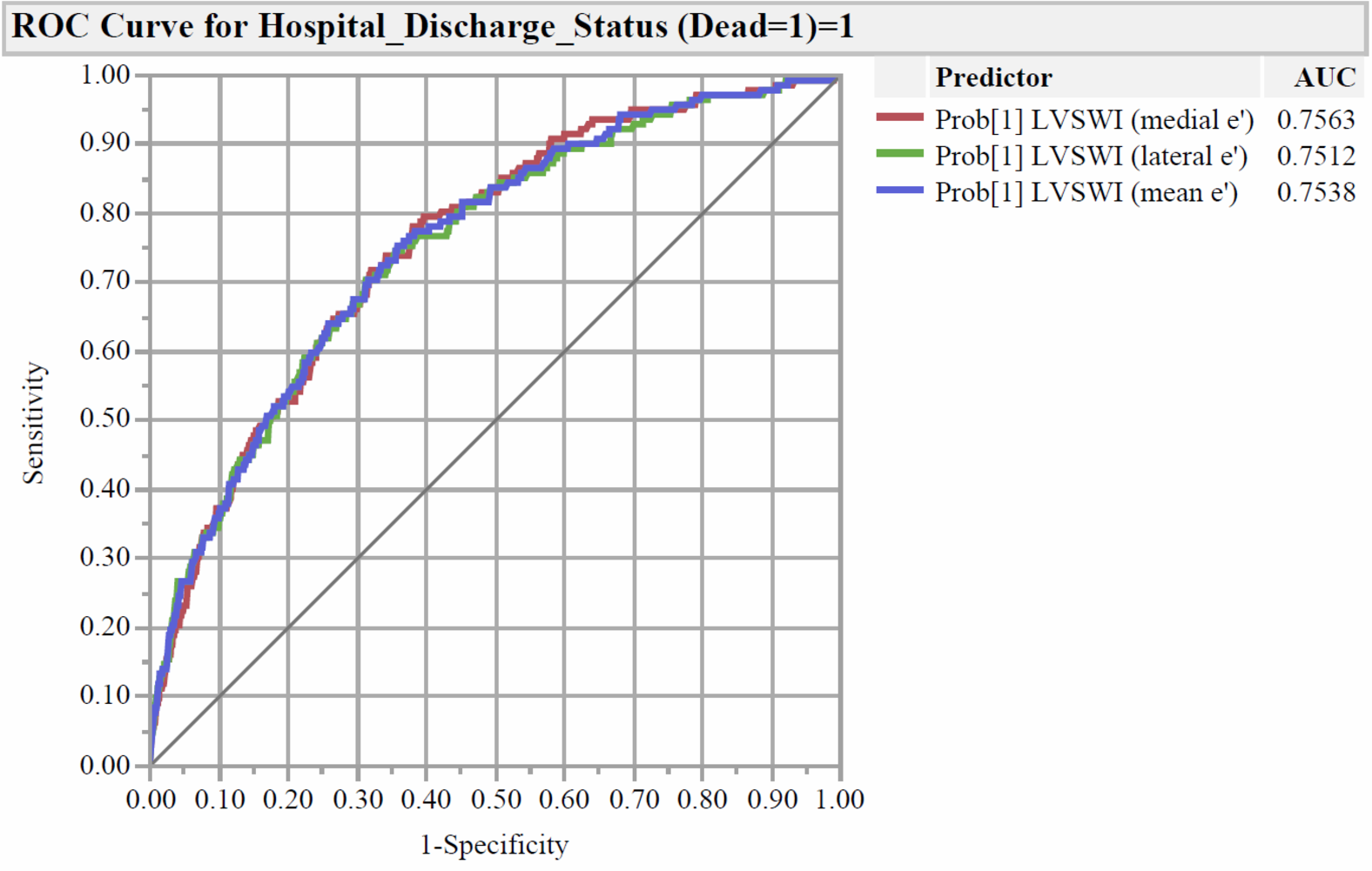

Supplement: S1 Fig — Receiver-operator characteristic (ROC) curves demonstrating discrimination of in-hospital mortality by ECHO-LVSWI calculated using the medial (red), lateral (green) or mean (blue) e’ velocity to estimate LVDEP for patients (n = 2896) with available data for both medial and lateral e’ velocity. P values for comparison of AUC values were all >0.05 by De Long test. (TIF) [file pone.0262053.s001.tif]

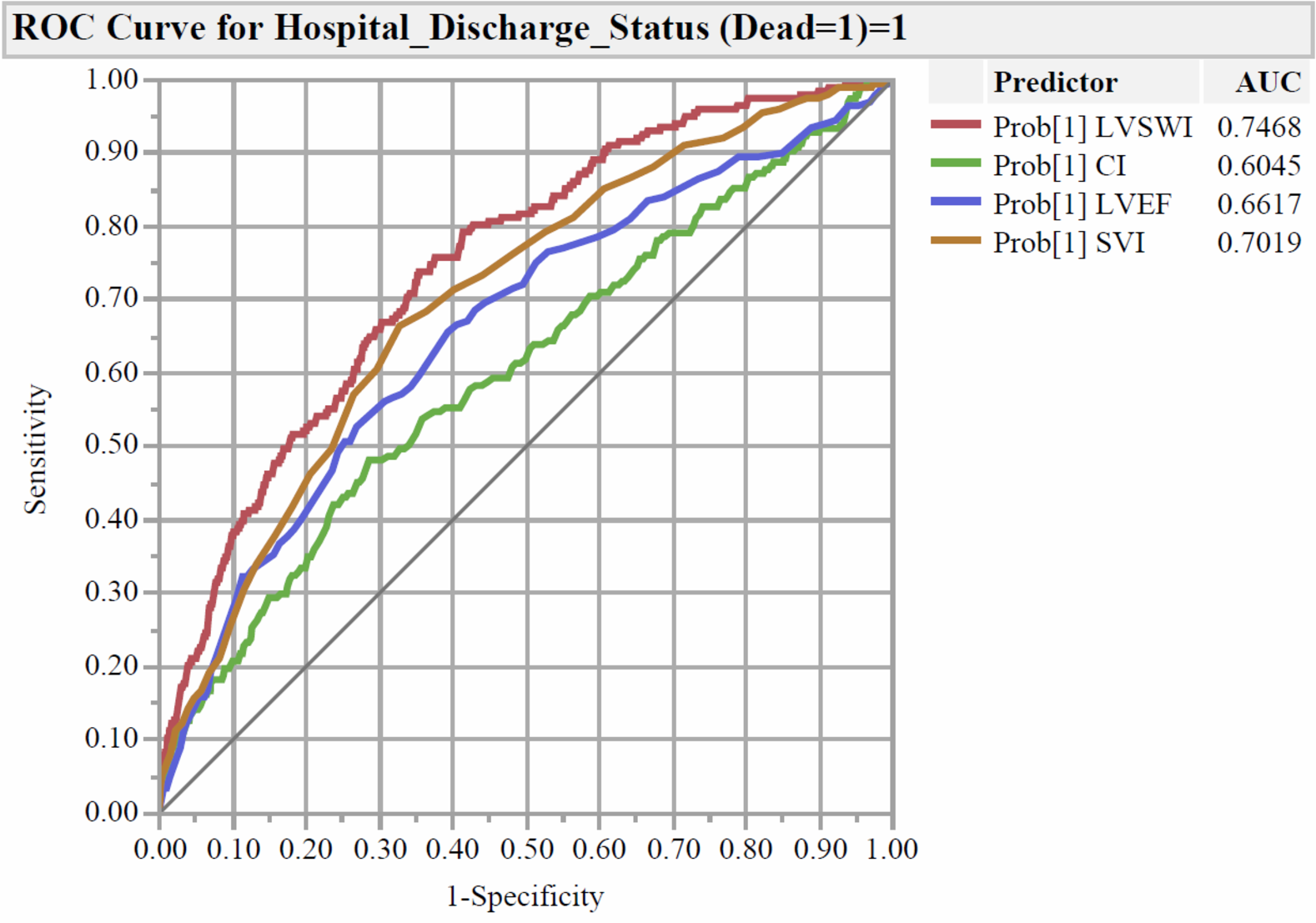

Supplement: S2 Fig — Receiver-operator characteristic (ROC) curves demonstrating discrimination of in-hospital mortality by ECHO-LVSWI (red), cardiac index (CI, green), LVEF (blue) and stroke volume index (SVI, orange). ECHO-LVSWI had a higher AUC value by the De Long test when compared with CI (p <0.0001), LVEF (p = 0.0002), or SVI (p = 0.06). (TIF) [file pone.0262053.s002.tif]

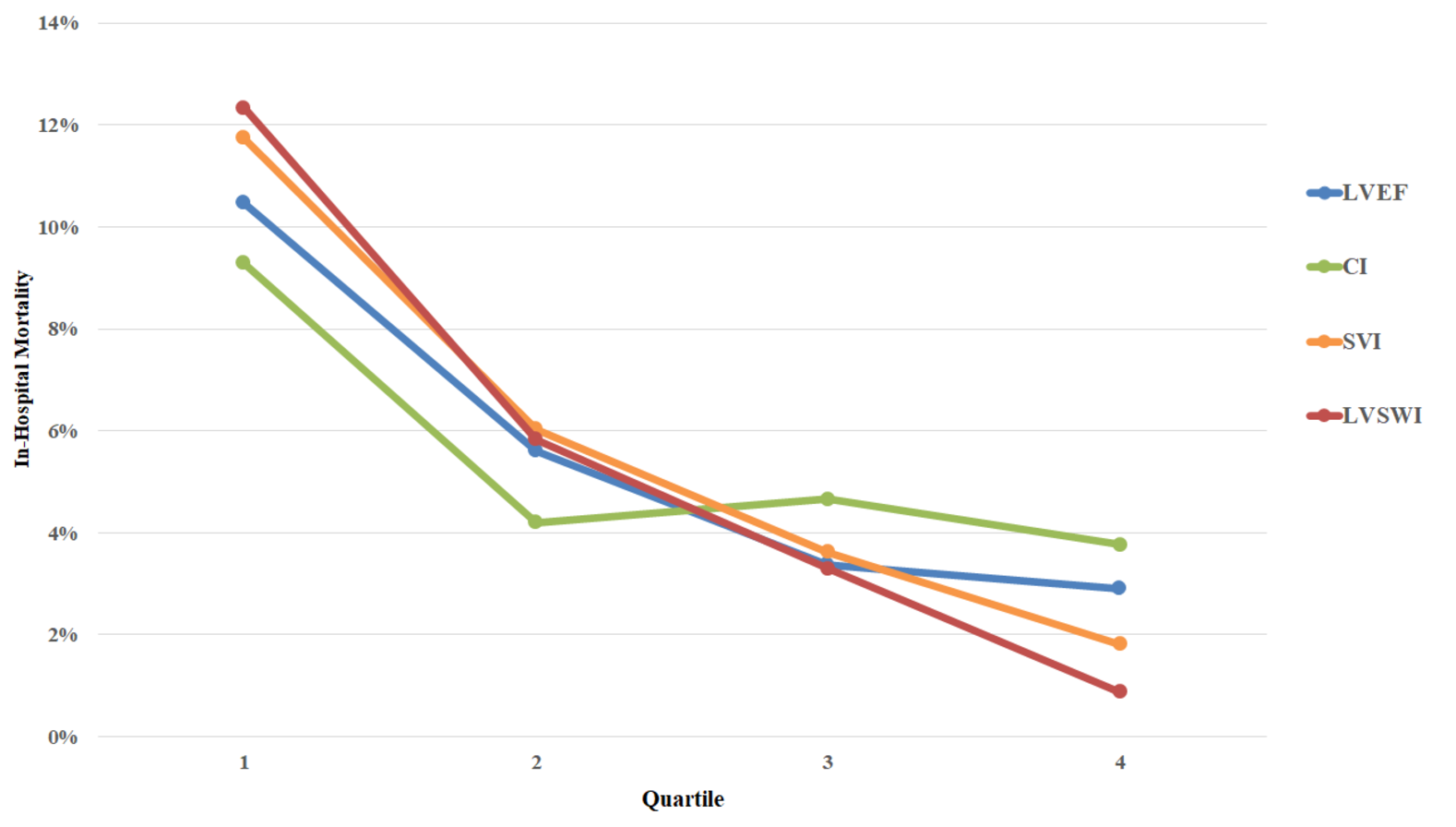

Supplement: S3 Fig — Observed in-hospital mortality in patients grouped by quartiles of ECHO-LVSWI (red), CI (green), LVEF (blue) and SVI (orange). Median and interquartile range values defining the quartiles are as follows: ECHO LVSWI, 37.0 (21.0, 46.1) J/m2; CI, 2.8 (2.4, 3.3) L/min/m2; LVEF, 51 (36, 61) %; SVI, 41 (33, 47) ml/m2. (TIF) [file pone.0262053.s003.tif]
