# Supplementary material for: Echocardiographic left ventricular stroke work index: An integrated noninvasive measure of shock severity
Source: PLoS One. 2022 Mar 9;17(3):e0262053. doi: 10.1371/journal.pone.0262053 (PMC8906587; doi:10.1371/journal.pone.0262053)
Supplement: S1 Table — (DOCX) [file pone.0262053.s004.docx]

**Echocardiographic left ventricular stroke work index: An integrated noninvasive measure of shock severity**

Jacob C. Jentzer, MD; Brandon M. Wiley, MD; Nandan S. Anavekar, MBBCh

From the Department of Cardiovascular Medicine, Mayo Clinic, Rochester, Minnesota

**S1 Table:** Measured and derived echocardiographic variables of interest.

| **Variable** |
| --- |
| Systolic blood pressure |
| Diastolic blood pressure |
| Heart rate |
| Heart rhythm (sinus rhythm or atrial fibrillation) |
| Left ventricular ejection fraction (LVEF) |
| Left ventricular outflow tract (LVOT) peak velocity |
| LVOT velocity-time integral (VTI) |
| Stroke volume (SV) |
| Stroke volume index (SVI) |
| Cardiac output (CO) |
| Cardiac index (CI) |
| Lateral mitral annulus peak systolic tissue Doppler (s’) velocity |
| Early mitral diastolic (E) velocity |
| Mitral atrial diastolic (A) velocity |
| Mitral E/A velocity ratio |
| Medial mitral annulus early diastolic tissue Doppler (e’) velocity |
| Medial mitral E/e’ velocity ratio |
| Lateral mitral annulus peak systolic tissue Doppler (s’) velocity |
| Mitral E wave deceleration time (DT) |
| Right atrial pressure (RAP) – estimated or measured |
| Left ventricular end-systolic dimension (LVESD) |
| Left ventricular end-diastolic dimension (LVEDD) |
| Left ventricular wall motion score index (WMSI) |
| Global right ventricular systolic function |
| Tricuspid annulus peak systolic tissue Doppler (s’) velocity |
| Peak tricuspid regurgitation (TR) systolic Doppler velocity |
