# Supplementary material for: Echocardiographic left ventricular stroke work index: An integrated noninvasive measure of shock severity
Source: PLoS One. 2022 Mar 9;17(3):e0262053. doi: 10.1371/journal.pone.0262053 (PMC8906587; doi:10.1371/journal.pone.0262053)
Supplement: S2 Table — (DOCX) [file pone.0262053.s005.docx]

**Echocardiographic left ventricular stroke work index: An integrated noninvasive measure of shock severity**

Jacob C. Jentzer, MD; Brandon M. Wiley, M; Nandan S. Anavekar, MBBCh

From the Department of Cardiovascular Medicine, Mayo Clinic, Rochester, Minnesota

**S2 Table:** Formulas used to calculate echocardiographic hemodynamic parameters, using data from the time of the echocardiogram.

| **Echocardiographic parameter** | **Formula** |
| --- | --- |
| Mean arterial pressure (MAP) | (SBP + 2 * DBP) / 3 |
| Stroke volume (SV) | (π/4) * LVOT VTI * (LVOT diameter)^2^ |
| Stroke volume index (SVI) | SV / BSA |
| Cardiac output (CO) | SV * HR (at time of LVOT VTI acquisition) |
| Cardiac index (CI) | CO / BSA or SVI * HR |
| Cardiac power output | (CO * MAP) / 451 |
| Cardiac power index | (CI * MAP) / 451 |
| Systemic vascular resistance (SVR) | 80 * (MAP – RAP) / CO |
| Systemic vascular resistance index (SVRI) | 80 * (MAP – RAP) / CI |
| Fractional shortening (FS) | (LVEDD – LVESD) / LVEDD |
| Pressure-adjusted heart rate (PAHR) | (HR * RAP) / MAP |
| Right ventricular systolic pressure (RVSP) | RAP + 4 * TR velocity^2^ |
| Myocardial contraction fraction | SV / LV myocardial volume |
| Left ventricular end-diastolic pressure (LVEDP)* | 4.9 + (0.62 * mitral E/e’ velocity ratio) |
| Left ventricular stroke work (LVSW)* | 0.0136 * [SV * (MAP – LVEDP)] |
| Left ventricular stroke work index (LVSWI)* | 0.0136 * [SVI * (MAP – LVEDP)] |

* From Choi, et al. Echocardiography 2010;27:1161-9.

Abbreviations: BSA, body surface area; DBP, diastolic blood pressure; HR, heart rate; LVOT, left ventricular outflow tract; RAP, right atrial pressure; SBP, systolic blood pressure; VTI, velocity-time integral. **See S1 Table for additional abbreviations.**
