# Supplementary material for: Echocardiographic left ventricular stroke work index: An integrated noninvasive measure of shock severity
Source: PLoS One. 2022 Mar 9;17(3):e0262053. doi: 10.1371/journal.pone.0262053 (PMC8906587; doi:10.1371/journal.pone.0262053)
Supplement: S4 Table — (DOCX) [file pone.0262053.s007.docx]

**Echocardiographic left ventricular stroke work index: An integrated noninvasive measure of shock severity**

Jacob C. Jentzer, MD; Brandon M. Wiley, M; Nandan S. Anavekar, MBBCh

From the Department of Cardiovascular Medicine, Mayo Clinic, Rochester, Minnesota

**S4 Table:**  Study definitions of Society for Cardiovascular Angiography and Intervention shock stages, as defined by Jentzer, et al. J Am Coll Cardiol 2019.

| **SCAI shock stage** | **Definition*** |
| --- | --- |
| **Stage A (At risk)** | NO criteria for **HYPOTENSION/TACHYCARDIA** or **HYPOPERFUSION** |
| **Stage B (Beginning)** | One or more criteria for **HYPOTENSION/TACHYCARDIA** but NO criteria for **HYPOPERFUSION** |
| **Stage C (Classic)** | One or more criteria for **HYPOPERFUSION** but NO criteria for **DETERIORATION** |
| **Stage D (Deteriorating)** | One or more criteria for **HYPOPERFUSION** and one or more criteria for **DETERIORATION** but NO criteria for **REFRACTORY** |
| **Stage E (Extremis)** | One or more criteria for **HYPOPERFUSION** and one or more criteria for **DETERIORATION** and one or more criteria for **REFRACTORY** |

*** See S3 Table for study definitions of HYPOTENSION/TACHYCARDIA, HYPOPERFUSION, DETERIORATION and REFRACTORY.**
