# Supplementary material for: Echocardiographic left ventricular stroke work index: An integrated noninvasive measure of shock severity
Source: PLoS One. 2022 Mar 9;17(3):e0262053. doi: 10.1371/journal.pone.0262053 (PMC8906587; doi:10.1371/journal.pone.0262053)
Supplement: Attachment — Submitted filename: Jentzer LVSWI SCAI stage Response to Reviewers 11-26-21.docx [file pone.0262053.s010.docx]

**Re: PONE-D-21-26906R1**

**“Echocardiographic left ventricular stroke work index: An integrated noninvasive measure of shock severity”**

**To:**

**Daniel A. Morris, M.D
Academic Editor
PLOS ONE

Dear Dr. Morris,**

**Thank you for considering this revised manuscript for publication in PLOS ONE. We have addressed the comments and requests to the best of our ability. We can clearly see the enthusiasm for a more extensive comparison of a variety of echocardiographic variables, but we want to emphasize that this study was designed with a rather limited scope—namely, to examine the ECHO-LVSWI in the context of the SCAI Shock classification. As such, extensive analyses of other echocardiographic variables outside of the structure of the SCAI Shock classification are not in line with our study hypothesis, and in some cases may impinge on our previously published works or distract from our main message. Therefore, in a few cases we have respectfully asked not to make the suggested changes even though we have tried to accommodate the suggested edits as much as possible. In addition, we have generated an online ECHO-LVSWI calculator to address concerns related to calculation time and arithmetic errors as a useful aid to clinicians who are interested in applying this variable. However, we are not able to hand-calculate ECHO-LVSWI from the primary echocardiographic images or using new patients based on the limits of our IRB approval. We hope that the Editor will understand these limitations and appreciate the efforts that we have made to address the remaining concerns and to ensure that this manuscript is a useful addition to the scientific literature. We appreciate your consideration.**

**Sincerely,**

**Jacob C. Jentzer, on behalf of the authors**

Editor Comments:

I would like to congratulate again to the authors for this excellent large study as well as for the effort to address all suggestions of the editors and reviewers. The manuscript is almost ready for publication, there are only some minor pending limitations that should be mandatorily addressed to get the final version of this interesting study/manuscript.

Pending Limitations and Comments:

1) The time-consuming and the potential high inter- or intra-observer variability of the LVSWI:
- The time-consuming to calculate the LVSWI is a serious issue that should be comprehensively addressed and discussed.
- The potential high inter- or intra-observer variability of the LVSWI is another potential issue of this index.

**Authors’ Response: We agree that the ECHO-LVSWI has important limitations in current practice related to the challenges in measuring and calculating ECHO-LVSWI at bedside. We have added a subheading “Limitations of ECHO-LVSWI” and amended the prior discussion to emphasize these points.**

**Manuscript excerpt:**

**“Calculating the ECHO-LVSWI by hand at bedside is time-consuming and prone to errors, including the potential for intra- and intra-observer variability, measurement inaccuracy and arithmetic errors during hand calculation. Therefore, it would be better to use an online calculator or ideally the ECHO-LVSWI could be calculated by the echocardiography imaging package or reporting software automatically. To facilitate use of ECHO-LVSWI, we have created an online calculator that can be used at the bedside (Supplemental X). Until automatic calculation of ECHO-LVSWI is incorporated into Doppler hemodynamic packages of bedside ultrasound machines or echocardiographic reporting software, we suggest that ECHO-LVSWI be calculated primarily using data obtained from a formal TTE to mitigate against these issues.”**

- Accordingly, the authors should analyze in at least 20 patients the time-consuming of the LVSWI as well as the inter- und/or intra-observer variability of this index (i.e., the absolute inter- und/or intra-observer mean differences of the LVSWI in at least 20 patients).

**Authors’ Response: We understand this request, but such an analysis extends beyond our IRB which covers only retrospective data gathering and analysis from the medical record, and not reviewing the echocardiographic images directly or collecting new patients prospectively. In this study, we calculated ECHO-LVSWI using data from formal TTE reports, and we advocate for using this approach in clinical practice at the current time. However, to try and assist providers in calculating the ECHO-LVSWI we have created formulas in an Excel spreadsheet as an online calculator, and this will help to facilitate the process and mitigate against arithmetic errors for end users. Using this calculator allows quick and accurate determination of ECHO-LVSWI and other hemodynamic calculations once the necessary data are measured (9 variables). We feel that providing this calculator for readers will be an important contribution, as we collaborate with imaging device companies to incorporate ECHO-LVSWI calculations into bedside ultrasound packages.**

**Manuscript excerpt:**

**“Calculating the ECHO-LVSWI by hand at bedside is time-consuming and prone to errors, including the potential for intra- and intra-observer variability, measurement inaccuracy and arithmetic errors during hand calculation. Therefore, it would be better to use an online calculator or ideally the ECHO-LVSWI could be calculated by the echocardiography imaging package or reporting software automatically. To facilitate use of ECHO-LVSWI, we have created an online calculator that can be used at the bedside (Supplemental File). Until automatic calculation of ECHO-LVSWI is incorporated into Doppler hemodynamic packages of bedside ultrasound machines or echocardiographic reporting software, we suggest that ECHO-LVSWI be calculated primarily using data obtained from a formal TTE to mitigate against these issues.”**

- In addition, please provide fundaments and discuss why we should measure or use the LSWI instead LVEF, SVi, or CI. By the way, please discuss in which scenario we should add the LSWI to conventional systolic parameters (for instance, in those with septic shock and HR < 90 beats/min…?).

**Authors’ Response: Our prior work has demonstrated that the LVEF and CI are inferior to SVI, which we have again shown using ROC analysis in this study (Supplemental Figure 3 as well as Supplemental Figure 4). We have likewise shown that LVSWI is superior to SVI, although this difference is of marginal significance (p = 0.06). We added these data to the prior revision. In addition, we have emphasized this point in the Discussion. At this time, it is premature to state that we know definitively which specific clinical scenarios call for use of ECHO-LVSWI as opposed to a different measurement, and our study was not designed to specifically answer this question. While we certainly understand the request to highlight the clinical value of the ECHO-LVSWI in this way, we do not want to make statements that are not supported by our analyses. This study was designed to establish the relevance of ECHO-LVSWI in the context of the SCAI shock classification for mortality risk stratification. Further research is clearly needed to extend the clinical utility of ECHO-LVSWI beyond risk stratification and into clinical decision-making.**

**Manuscript excerpt:**

**Results**

**“LVSWI had a higher AUC value (Supplemental Figure 3) than LVEF (AUC 0.662, p <0.001), cardiac index (AUC 0.604, p <0.001) and SVI (AUC 0.702, p = 0.06).** **When patients were grouped by quartiles of these variables, ECHO-LVSWI produced the greatest separation between high-risk and low-risk patients (Supplemental Figure 4 and Supplemental Table 5).”**

**Discussion**

**“We have demonstrated that ECHO-LVSWI has higher discrimination for in-hospital mortality than LVEF, SVI and CI when analyzed as continuous variables, providing risk stratification by separating high-risk and low-risk CICU patients.”**

**“ECHO-LVSWI may prove to be a superior non-invasive parameter because it integrates diastolic function assessment, providing deeper insights into overall myocardial function that translates into improved mortality risk stratification.”**

**“Further research is necessary to determine when the potentially laborious calculation of ECHO-LVSWI is necessary, as opposed to the less-predictive but simpler SVI itself.”**

2) The potential incremental value of the LSWI over conventional LV systolic parameters:
- The supplemental figures X and Y and the supplemental table 5 are excellent and thus, it should be included in the main manuscript. By the way, please provide cutoff of the Youden index and the sensitivity and specificity of the cutoffs of the LVSWI, CI, LVEF, and SVi. In addition, please provide the values of the quartiles of the LVSWI, LVEF, CI, and SVi from the supplemental table 5.

**Authors’ Response: We perceive the Reviewer’s strong interest in these comparisons between echocardiographic variables, yet we want to emphasize that this study was not designed to simply validate the ECHO-LVSWI in comparison to other TTE variables, but rather to examine the ECHO-LVSWI in the context of shock severity defined by SCAI shock stage (and not as a standalone risk stratification tool). Our prior work has examined LVSWI as a standalone risk stratification tool and has explored LVEF, SVI and CI in the context of SCAI shock stage. We worry that these specific figures and tables might distract from the primary message. As such, we feel that these supplemental figures (which are not related to SCAI shock stage) are tangential to the main hypothesis and respectfully request that they remain supplemental for this reason; if the Editor feels strongly, we can include them as main figures. As requested, we have added the median and IQR values (which define the quartiles) to the figure legends and have added a Supplemental Table 6 with the requested diagnostic performance of the echocardiographic variables.
Manuscript excerpt:**

**Results**

**“At the optimal cut-off, LVSWI had the highest combined sensitivity and specificity (Supplemental Table 6).”**
