# Supplementary material for: Echocardiographic left ventricular stroke work index: An integrated noninvasive measure of shock severity
Source: PLoS One. 2022 Mar 9;17(3):e0262053. doi: 10.1371/journal.pone.0262053 (PMC8906587; doi:10.1371/journal.pone.0262053)
Supplement: Attachment — Submitted filename: Jentzer LVSWI SCAI stage Response to Reviewers 12-1-21.docx [file pone.0262053.s011.docx]

**Re: PONE-D-21-26906R2**

**“Echocardiographic left ventricular stroke work index: An integrated noninvasive measure of shock severity”**

**To:**

**Daniel A. Morris, M.D
Academic Editor
PLOS ONE

Dear Dr. Morris,**

**Thank you for considering this revised manuscript for publication in PLOS ONE. We have addressed all the comments and requests as instructed. We hope that you will find this revised manuscript to be acceptable for publication.**

**Sincerely,**

**Jacob C. Jentzer, on behalf of the authors**

**---------------------------------------------------------------------------------------------------------**

Additional Editor Comments:

Thank you very much again for the efforts to address the pending limitations from this study. While the revised version has improved, it remains some limitations that should be mandatorily addressed to get priority for publishing this study in PlosOne.

Pending Limitations:

1) Please highlight in the limitations section that “the echocardiographic data was merely obtained from medical records. Accordingly, it remains uncertain what would be the time-consuming and the inter- and intra-observer variability of the LVSWI”.

**Authors’ Response: This suggested addition has been made, although we have used our own wording.**

**Manuscript excerpt:**

**Discussion**

**“We calculated ECHO-LVSWI automatically using data extracted from formal TTE reports in the medical record, as opposed to manual review of the primary TTE images. Calculating the ECHO-LVSWI by hand at bedside is time-consuming and prone to errors, including the potential for intra- and intra-observer variability, measurement inaccuracy and arithmetic errors during hand calculation. Accordingly, the time demand necessary to manually calculate the ECHO-LVSWI and the extent to which the inter- and intra-observer variability of manually calculated ECHO-LVSWI might influence its observed association with mortality remain uncertain.”**

2) The following figures and tables should be completed and mandatorily incorporated into the main manuscript to improve the presentation of this study:
- Supplemental Figures 1a and 1b.
- Supplemental Table 5 (please add the values of the quartiles in this table).
- Supplemental Table 6 (please delete the file “Youden’s J index @ cut-off” in this table).

**Authors’ Response: These suggested additions have been made, and the tables and figures have been renumbered.**
